# Fully Automated Biometric Parameter Measurement in Prenatal Ultrasound Screening for Total Anomalous Pulmonary Venous Connection

**DOI:** 10.3390/bioengineering13070822

**Published:** 2026-07-17

**Authors:** Rina Aoyama, Naoaki Harada, Masaaki Komatsu, Reina Komatsu, Katsuji Takeda, Naoki Teraya, Ken Asada, Syuzo Kaneko, Kazuki Iwamoto, Ryu Matsuoka, Akihiko Sekizawa, Ryuji Hamamoto

**Affiliations:** 1 Department of Obstetrics and Gynecology, Showa Medical University School of Medicine, 1-5-8 Hatanodai, Shinagawa-ku, Tokyo 142-8666, Japan; rina.a1013@med.showa-u.ac.jp (R.A.); rkomatsu@med.showa-u.ac.jp (R.K.); ryu@med.showa-u.ac.jp (R.M.); sekizawa@med.showa-u.ac.jp (A.S.); 2 Department of NCC Cancer Science, Biomedical Science and Engineering Track, Graduate School of Medical and Dental Sciences, Institute of Science Tokyo, 1-5-45 Yushima, Bunkyo-ku, Tokyo 113-8510, Japan; naharad@ncc.go.jp (N.H.); katake2@ncc.go.jp (K.T.); 3 Division of Medical AI Research and Development, National Cancer Center Research Institute, 5-1-1 Tsukiji, Chuo-ku, Tokyo 104-0045, Japan; ken.asada@riken.jp (K.A.); sykaneko@ncc.go.jp (S.K.); 4 Digital Health Platform Development Office, Healthcare Business Unit, Fujitsu Japan Ltd., 1-5 Omiya-cho, Saiwai-ku, Kawasaki 212-0014, Japan; iwamoto.kazuki@fujitsu.com; 5 AI Medical Engineering Team, RIKEN Center for Advanced Intelligence Project, 1-4-1 Nihonbashi, Chuo-ku, Tokyo 103-0027, Japan

**Keywords:** total anomalous pulmonary venous connection, prenatal ultrasound screening, post-left atrium space index, left-atrial posterior-space-to-diagonal ratio, artificial intelligence, fetal echocardiography

## Abstract

Total anomalous pulmonary venous connection (TAPVC) is a severe congenital heart disease, yet its prenatal detection rate remains suboptimal. To support prenatal ultrasound screening of TAPVC, the post-left atrium space (PLAS) index and the left-atrial posterior-space-to-diagonal (LAPSD) ratio measured in the four-chamber view (4CV) have been proposed as useful biometric parameters. In this study, we developed a novel approach that integrates automated 4CV extraction (AE) from fetal cardiac ultrasound videos with automated measurement of these indices. The heart, crux, and descending aorta were segmented using DeepLabv3+, UNet3+, and SegFormer. The screening performance of the AE-based methods was comparable to that of manual 4CV extraction, as demonstrated by similar mean areas under the receiver operating characteristic curve (AUCs). In a clinical comparison study, the mean AUC values for residents, fellows, experts, AE-DeepLabv3+, AE-UNet3+, and AE-SegFormer were 0.784, 0.801, 0.996, 0.903, 0.928, and 0.940, respectively, for the PLAS index and 0.797, 0.801, 0.996, 0.919, 0.916, and 0.940, respectively, for the LAPSD ratio. Although experts demonstrated the best overall performance, the fully automated methods consistently outperformed both the residents and fellows. This approach may support less experienced examiners, improve screening accuracy, streamline clinical workflows, and ultimately enhance the prenatal detection of TAPVC.

## 1. Introduction

Congenital heart disease (CHD) affects approximately 1% of fetuses worldwide [1,2]. Disease severity ranges from mild conditions that resolve spontaneously to critical defects that cause cyanosis shortly after birth and require immediate medical intervention. Despite the clinical importance of early diagnosis, the global prenatal detection rate for CHD has historically remained suboptimal, ranging from 50% to 70%, with considerable variation among specific cardiac defects [3]. In addition, substantial regional differences in prenatal detection rates have been reported [4].

Total anomalous pulmonary venous connection (TAPVC) is a severe form of CHD that can lead to sudden death. According to the 2023 Japanese survey of childhood heart diseases, 10,975 cases of CHD (1.5% of all births) and 141 cases of TAPVC (1.3% of all CHD cases) were identified among 727,277 births [5]. TAPVC is a structural cardiac anomaly in which the pulmonary veins drain into the systemic venous circulation rather than the left atrium [6]. This abnormality results in hypoxemia and pulmonary hypertension in infants. Mortality remains high even during the perinatal period, with one report indicating that 81.5% of affected patients died without treatment [7]. Surgical correction is the only definitive treatment, and only a limited number of centers are equipped to perform this procedure [8]. In Japan, outcomes for TAPVC have improved substantially over the past 25 years, with a reported 10-year survival rate of approximately 90% and an operative mortality rate of about 0.7% [9,10]. Consequently, prenatal diagnosis is essential to facilitate appropriate planning of the delivery location, timing, and mode of delivery, as well as postnatal management and surgical treatment [11].

Prenatal ultrasound screening for TAPVC relies on several characteristic findings, including the absence of pulmonary venous connection to the left atrium, ventricular asymmetry with a larger right ventricle than left ventricle, a small left atrium, and an increased distance between the descending aorta and the posterior wall of the left atrium. Despite these recognized features, the prenatal detection rate of TAPVC remains low, ranging from 1.9% to 10%, highlighting the need for improved diagnostic approaches [12,13]. To facilitate prenatal ultrasound screening, the post-left atrium space (PLAS) index and the left-atrial posterior-space-to-diagonal (LAPSD) ratio measured on the four-chamber view (4CV) have been proposed as biometric parameters [7,14]. These indices are reportedly independent of fetal sex, gestational age, and body size and demonstrate high sensitivity and specificity. However, their measurement currently relies on manual assessment, which is time-consuming and subject to inter- and intra-observer variability. In addition, the quality of fetal cardiac ultrasound images depends heavily on the experience and technical expertise of the examiner [15]. A retrospective study demonstrated significant differences in fetal cardiac ultrasound image quality between cases with prenatally detected CHD and those with missed diagnoses [16].

In recent years, artificial intelligence (AI) technologies have been increasingly adopted across a wide range of medical specialties [17,18,19]. Advances have occurred not only in research but also in clinical implementation [20,21]. As of March 2026, the U.S. Food and Drug Administration database included 1,430 approved AI-enabled medical devices [22]. However, even after regulatory approval and commercialization, the effectiveness and generalizability of AI-enabled medical devices require validation through prospective clinical studies [23].

The integration of AI into fetal cardiac ultrasound assessment may improve the objectivity, consistency, and accuracy of biometric measurements [24]. In this study, we developed a novel fully automated method for identifying appropriate 4CVs from fetal cardiac ultrasound videos and calculating the PLAS index and LAPSD ratio. We also conducted a clinical comparison study to evaluate the performance of the proposed AI-based approach against that of obstetricians.

## 2. Materials and Methods

### 2.1. Data Preparation

Fetal cardiac ultrasound videos from 203 singleton fetuses were included in this study. All fetuses underwent mid-trimester fetal cardiac ultrasound screening at Showa Medical University Hospitals in Tokyo, Japan. The cohort comprised 172 normal cases, 25 cases with CHD other than TAPVC, and six cases with TAPVC. The mean gestational age was 21 weeks (range, 18–34 weeks) in the normal group, 23 weeks (range, 19–35 weeks) in the non-TAPVC CHD group, and 24 weeks (range, 23–31 weeks) in the TAPVC group.

The non-TAPVC CHD group included cases of tetralogy of Fallot, complete transposition of the great arteries, ventricular septal defect, atrial septal defect, pulmonary stenosis, pulmonary atresia with intact ventricular septum, tricuspid insufficiency, double-outlet right ventricle, persistent left superior vena cava, and Ebstein anomaly. Multiple cardiac anomalies were present in some cases.

Ultrasound examinations were performed by specialists certified in fetal echocardiography or by obstetricians under their supervision using Voluson^®^ E8 or E10 systems (GE Healthcare, Chicago, IL, USA) in accordance with established guidelines [25]. Each ultrasound video captured a continuous sequence of cross-sectional images extending from the fetal stomach to the aortic arch.

### 2.2. Data Preprocessing and Augmentation

For manual 4CV extraction (ME), an appropriate 4CV was manually selected from each ultrasound video by an obstetrician under the supervision of two fetal echocardiography experts. The obstetrician generated pixel-level annotations of the heart, crux, and descending aorta, and all annotations were reviewed and validated by the supervising experts. For model development, 400 images from 130 normal cases were randomly selected for training, and 100 images from 32 normal cases were used for testing. In addition, 100 images from 25 non-TAPVC CHD cases and 28 images from six TAPVC cases were included in the test set. The training data were further divided into training and validation subsets at a ratio of 4:1. No case was included in more than one dataset, ensuring complete separation among the training, validation, and test sets. To perform cross-validation, five independent training–test splits were created such that all normal cases were included in the test set at least once.

To compare different extraction approaches, we developed an automated 4CV extraction (AE) method using the object detection model YOLOv7 [26]. As described in our previous study, an appropriate 4CV was automatically selected when the following cardiac structures were simultaneously detected at their predefined confidence thresholds: the crux, right atrium, right ventricle, left atrium, left ventricle, and descending aorta [27]. To balance the dataset, the number of extracted 4CV images was limited to two per video for normal cases and ten per video for non-TAPVC CHD cases, whereas no limit was imposed for TAPVC cases. The final AE dataset comprised 512 images from normal cases, 324 images from non-TAPVC CHD cases, and 153 images from TAPVC cases.

All extracted 4CV images were subsequently cropped using YOLOv7, regardless of the extraction method. Cropping was performed using an object detection approach based on bounding boxes. Specifically, the minimum and maximum x- and y-coordinates of all detected structures within an image were identified. The cropping boundaries were then defined by extending the minimum and maximum coordinates by five pixels in each direction, thereby expanding the cropped region by five pixels on all sides.

Because of the limited dataset size, data augmentation was applied to the training images. Augmentation included random adjustments to image rotation, brightness, and contrast. Image rotation was performed within a range of ±15°. Brightness and contrast were adjusted according to the following equation, where src represents the original image and dst represents the augmented image:

dst (I) = saturate_cast (|src(I) × α + β|).(1)

The value of α ranged from 0.7 to 1.3, whereas the value of β ranged from −30 to 30.

### 2.3. Segmentation

The heart, crux, and descending aorta were segmented from the extracted 4CV images using DeepLabv3+ [28], UNet3+ [29], and SegFormer [30]. Hyperparameters for each model were selected based on previous studies, and ImageNet pre-training was applied to DeepLabv3+ and SegFormer. Segmentation performance was evaluated using the Dice coefficient, which was calculated from the numbers of true positive (TP), false positive (FP), and false negative (FN) cases as follows:

Dice = 2TP/(2TP + FP + FN).(2)

The Dice coefficient ranges from 0 to 1, with values closer to 1 indicating better segmentation performance. To compare the accuracy of the three segmentation models, the mean Dice coefficient (mDice) was calculated for each image by comparing the model predictions with the corresponding ground-truth labels.

### 2.4. Computation of the PLAS Index and the LAPSD Ratio

**Figure 1:**
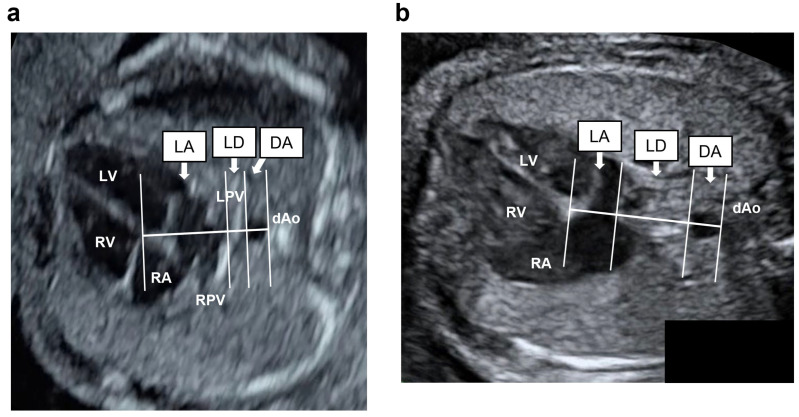
Assessment of the PLAS index and LAPSD ratio on the four-chamber view in a normal case (**a**) and a case of TAPVC (**b**). Both biometric parameters are based on the characteristic ultrasound finding that LD is wider in TAPVC (**b**) than in the normal case (**a**). PLAS index, post-left atrium space index; LAPSD ratio, left-atrial posterior-space-to-diagonal ratio; LA, crux–posterior left atrial wall distance; LD, posterior left atrial wall–descending aorta distance; DA, diameter of the descending aorta; LV, left ventricle; RV, right ventricle; RA, right atrium; LPV, left pulmonary vein; RPV, right pulmonary vein; dAo, descending aorta.

In TAPVC, the pulmonary veins do not connect to the left atrium, resulting in a characteristic widening of LD on fetal ultrasound. Consequently, both the PLAS index and the LAPSD ratio have been reported to be significantly higher in TAPVC cases than in normal fetuses and those with other forms of CHD. Based on previous studies, the normal range in the present study was defined as a PLAS index < 1 and a LAPSD ratio < 0.35. Mean values for both indices remained within the normal range in normal and non-TAPVC CHD cases, whereas mean values in TAPVC cases exceeded these thresholds (Figure 1) [7,14,31,32].

To obtain these measurements, the program first calculated the centroids of the annotated or segmented crux and descending aorta. A straight line was then drawn through the centroids of these two structures. The coordinates of the intersections between this line and the boundaries of the heart and descending aorta were identified. Distances were subsequently calculated from these intersection points (Supplementary Figure S1).

**Figure 2:**
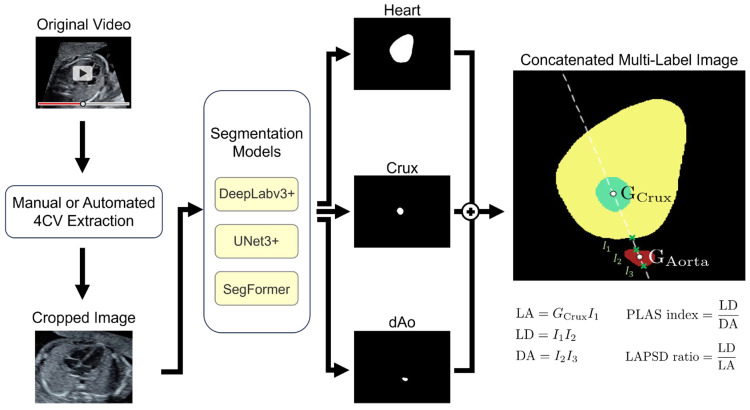
Workflow of biometric parameter measurement. Following manual or automated extraction of a predefined number of 4CV images, the heart, crux, and descending aorta are segmented using artificial intelligence models. The PLAS index and LAPSD ratio are subsequently calculated based on the segmentation results. The dashed line represents the line connecting the centroids of the crux and descending aorta. Green crosses indicate the intersection points I_1_, I_2_, and I_3_. The differently colored regions represent the segmented heart, crux, and descending aorta. PLAS index, post-left atrium space index; LAPSD ratio, left-atrial posterior-space-to-diagonal ratio; 4CV, four-chamber view; dAo, descending aorta; G, centroid; I, intersection point; LA, crux–posterior left atrial wall distance; LD, posterior left atrial wall–descending aorta distance; DA, diameter of the descending aorta.

The PLAS index and LAPSD ratio were calculated as follows:

(3) $$PLAS\;index\;=\;\frac{LD}{DA}\;LAPSD\;ratio\;=\;\frac{LD}{LA}\;$$

where *LA* denotes the distance between the crux and the posterior wall of the left atrium, *LD* denotes the distance between the posterior wall of the left atrium and the descending aorta, and *DA* denotes the diameter of the descending aorta. The values of *LA*, *LD*, and *DA* were derived from the segmentation results using the following procedure. First, the centroid coordinates $\left(\bar{x},\;\bar{y}\right)$ of each segmented region *D* were calculated as follows:

(4) $$\bar{x}\;=\;\frac{\sum_{x,y\in D}v_{xy}x}{\sum_{x,y\in D}v_{xy}}\;\bar{y}\;=\;\frac{\sum_{x,y\in D}v_{xy}y}{\sum_{x,y\in D}v_{xy}}$$

where $v_{xy}$ denotes the pixel value at coordinate (*x*, *y*). Next, the intersection points $I_{1}$, $I_{2}$, and $I_{3}$ between the line connecting the centroids of the crux ($G_{Crux})$ and descending aorta ($G_{Aorta})$ and the boundaries of the left atrium and descending aorta were identified. The intersection point on the descending aorta closest to $G_{Crux}$ was designated as $I_{2}$. Finally, the lengths of the line segments $G_{Crux}I_{1}$, $I_{1}I_{2}$, and $I_{2}I_{3}$ were calculated and defined as *LA*, *LD*, and *DA*, respectively (Figure 2).

To evaluate TAPVC screening performance, receiver operating characteristic (ROC) curve and precision-recall (PR) curve analyses were performed for both the PLAS index and LAPSD ratio using six different approaches, comprising the combination of two extraction methods (ME and AE) and three segmentation models (DeepLabv3+, UNet3+, and SegFormer). The test dataset included 63 fetal ultrasound videos, with one video obtained from each case, including 32 normal cases, 25 non-TAPVC CHD cases, and six TAPVC cases. The screening performance of each method was assessed using the area under the ROC and PR curves (AUC). AUC values were calculated for both the PLAS index and LAPSD ratio, and the arithmetic mean of the AUC values was used for comparison among methods. Higher AUC values indicate greater accuracy in distinguishing TAPVC cases from normal and non-TAPVC CHD cases.

### 2.5. Clinical Comparison Study

A clinical comparison study was conducted to evaluate the performance of obstetricians and the proposed fully automated methods. The study included three experts, eight fellows, and nine residents from Showa Medical University Hospitals. The experts were certified in fetal echocardiography, the fellows were obstetricians with at least three years of experience, and the residents had less than three years of experience.

All participants were provided with a test dataset consisting of 20 fetal ultrasound videos, with one video obtained from each case, including 10 normal cases, seven non-TAPVC CHD cases, and three TAPVC cases. To replicate routine clinical practice, participants were first asked to manually select a single ultrasound image from each video that contained the optimal 4CV for measurement of the PLAS index and LAPSD ratio.

Participants then drew a straight line extending from the crux through the descending aorta, followed by perpendicular lines at the crux, the posterior wall of the left atrium, and the ventral and dorsal borders of the descending aorta. The coordinates of the resulting four intersection points were automatically identified by the program, which subsequently calculated the PLAS index and LAPSD ratio (Supplementary Figure S2).

The three fully automated methods were implemented according to the procedures described in Section 2.2, Section 2.3 and Section 2.4. TAPVC screening performance was then compared across six groups: experts, fellows, residents, AE-DeepLabv3+, AE-UNet3+, and AE-SegFormer. Performance was evaluated using the ROC and PR curve analyses and corresponding AUC values.

### 2.6. Software and Computational Environment

All automated image-processing procedures, including 4CV extraction, segmentation, and calculation of the PLAS index and LAPSD ratio, were performed using Python 3.8.12, PyTorch 1.13.0, OpenCV 4.6.0, and CUDA 11.7. Statistical analyses, including ROC and PR curve analyses, were performed using Python 3.13.1 with scikit-learn 1.6.1, SciPy 1.15.1, and pandas 2.2.3. The corresponding figures were generated using Matplotlib 3.10.0.

## 3. Results

### 3.1. Segmentation Performance

**Figure 3:**
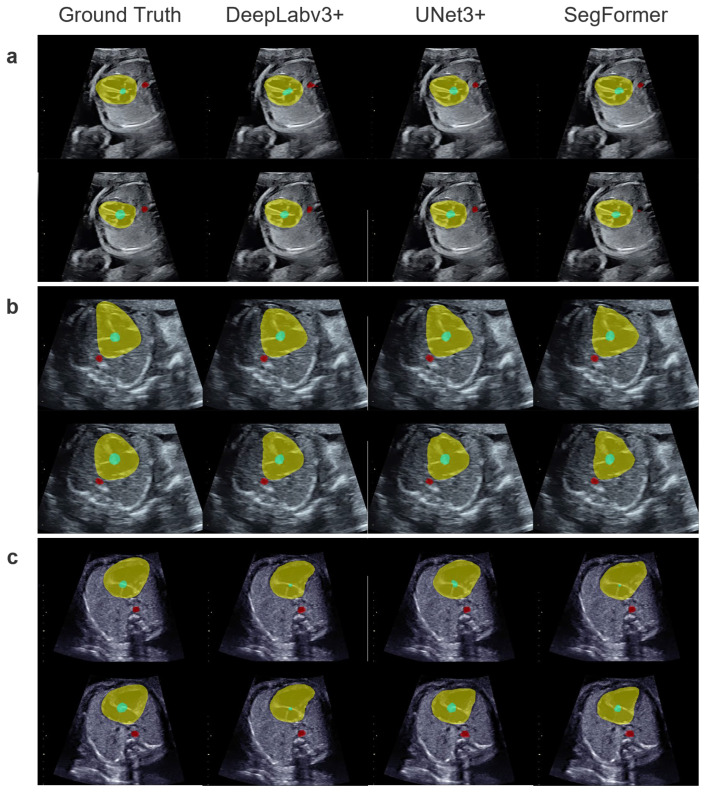
Representative segmentation results on four-chamber view images from a normal case (**a**), a non-TAPVC CHD case (**b**), and a TAPVC case (**c**). The leftmost images show the ground-truth annotations. Each horizontal row presents the segmentation results generated by a different model for the same case. Within each case, the upper images were manually extracted, whereas the lower images were automatically extracted using YOLOv7. The heart is shown in yellow, the crux in light green, and the descending aorta in red. TAPVC, total anomalous pulmonary venous connection; CHD, congenital heart disease.

Figure 3 presents representative segmented 4CV images generated by the six methods for a normal case (a), a non-TAPVC CHD case (b), and a TAPVC case (c). Visual comparison revealed no apparent differences between ME images shown in the upper panels and AE images shown in the lower panels. All methods successfully captured the characteristic TAPVC feature of an increased LD compared with normal and non-TAPVC CHD cases. In addition, the segmented crux region varied among the three segmentation models, particularly in the TAPVC case.

Table 1 summarizes the quantitative segmentation performance of the six methods using mDice. Across all three segmentation models, mDice values showed a gradual decline from normal cases to non-TAPVC CHD cases and TAPVC cases. Among the segmented structures, the crux consistently exhibited the lowest segmentation performance. Overall, segmentation accuracy was comparable between ME and AE images. Among the three segmentation models, SegFormer achieved the highest overall performance, although DeepLabv3+ provided the most accurate segmentation of the descending aorta in certain case groups.

### 3.2. TAPVC Screening Performance Comparison Between ME and AE

**Figure 4:**
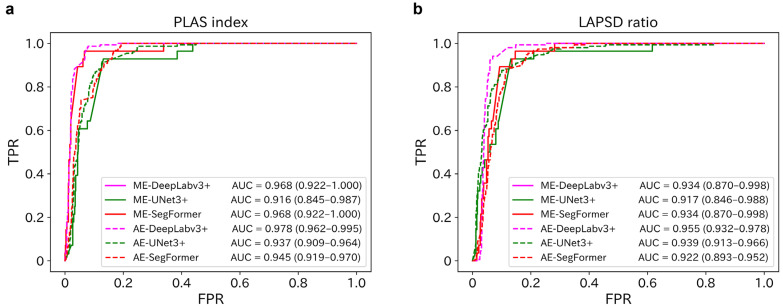
TAPVC screening performance of the segmentation models using ME and AE based on the PLAS index (**a**) and LAPSD ratio (**b**). ROC curves are shown for each method. Mean AUC values with 95% confidence intervals are provided in the legends. ROC, receiver operating characteristic; AUC, area under the receiver operating characteristic curve; TPR, true-positive rate; FPR, false-positive rate; AE, automated extraction; ME, manual extraction; PLAS index, post-left atrium space index; LAPSD ratio, left-atrial posterior-space-to-diagonal ratio; TAPVC, total anomalous pulmonary venous connection.

Using the methods described above, the mean PLAS index and LAPSD ratio were calculated for the test dataset comprising 32 normal cases, 25 non-TAPVC CHD cases, and six TAPVC cases. TAPVC screening performance was then evaluated using ROC curve analysis. For the PLAS index, the mean AUC values for ME-DeepLabv3+, ME-UNet3+, ME-SegFormer, AE-DeepLabv3+, AE-UNet3+, and AE-SegFormer were 0.968, 0.916, 0.968, 0.978, 0.937, and 0.945, respectively. For the LAPSD ratio, the corresponding mean AUC values were 0.934, 0.917, 0.934, 0.955, 0.939, and 0.922, respectively. Among all methods, AE-DeepLabv3+ achieved the highest AUC values for both the PLAS index and LAPSD ratio (Figure 4). In the PR curve analysis, AE-DeepLabv3+ achieved the highest AUC value of 0.807 for the PLAS index, and AE-UNet3+ achieved a value of 0.688 for the LAPSD ratio (Supplementary Figure S3). Overall, the AE-based methods demonstrated a screening performance comparable to or superior to that of the ME-based methods.

### 3.3. TAPVC Screening Performance Comparison Between Obstetricians and Fully Automated Methods

**Figure 5:**
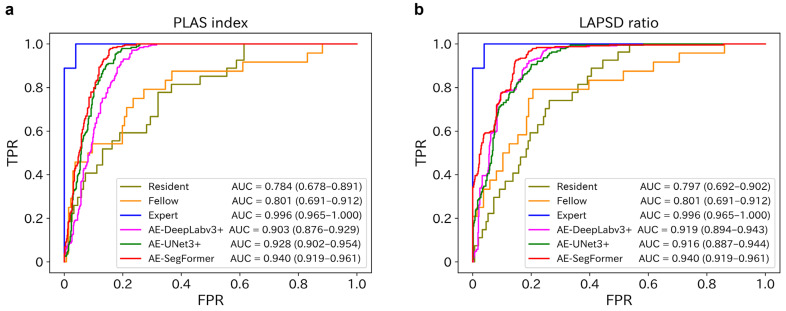
Comparison of TAPVC screening performance between obstetricians and the fully automated methods based on the PLAS index (**a**) and LAPSD ratio (**b**). ROC curves illustrate the screening performance of experts, fellows, residents, and the fully automated methods. Mean AUC values with 95% confidence intervals are provided in the legends. ROC, receiver operating characteristic; AUC, area under the receiver operating characteristic curve; TPR, true-positive rate; FPR, false-positive rate; AE, automated extraction; PLAS index, post-left atrium space index; LAPSD ratio, left-atrial posterior-space-to-diagonal ratio; TAPVC, total anomalous pulmonary venous connection.

Figure 5 compares TAPVC screening performance between obstetricians and the proposed fully automated methods using ROC curve analysis. For the PLAS index, the mean AUC values for residents, fellows, experts, AE-DeepLabv3+, AE-UNet3+, and AE-SegFormer were 0.784, 0.801, 0.996, 0.903, 0.928, and 0.940, respectively. For the LAPSD ratio, the corresponding mean AUC values were 0.797, 0.801, 0.996, 0.919, 0.916, and 0.940, respectively. In the PR curve analysis, the mean AUC values were 0.384, 0.453, 0.979, 0.690, 0.724, and 0.799, respectively, for the PLAS index. The corresponding mean AUC values were 0.339, 0.523, 0.979, 0.737, 0.756, and 0.862, respectively, for the LAPSD ratio (Supplementary Figure S4). Among the AE-based methods, AE-SegFormer achieved the highest AUC values for both indices in the ROC and PR curve analyses. Overall, experts demonstrated the best screening performance, but the AE-based methods consistently outperformed the residents and fellows. These findings suggest that the proposed automated methods may assist less experienced examiners by providing TAPVC screening performance approaching that of expert fetal echocardiographers.

## 4. Discussion

Numerous studies have demonstrated the benefits of prenatal diagnosis for CHD. One study reported a preoperative mortality rate of 0.5% among infants with neonatal heart disease diagnosed prenatally, compared with 3.2% among those diagnosed after birth [11]. Similarly, Colaco et al. reported comparable findings in low- and middle-income countries, with a mortality rate of 0.0% among prenatally diagnosed infants versus 5.6% among those diagnosed postnatally [33]. Prenatal identification of severe CHD allows parents to receive counseling and plan delivery at a specialized cardiac care center. This facilitates timely postnatal management and has been associated with lower preoperative neonatal mortality rates [34]. In the case of TAPVC, affected infants often present with right-sided pressure and volume overload and may be misdiagnosed as having persistent pulmonary hypertension of the newborn. However, the management of these two conditions differs substantially. Treatment for persistent pulmonary hypertension of the newborn typically includes tracheal intubation and administration of oxygen and nitric oxide. In infants with TAPVC, such interventions may exacerbate pulmonary venous obstruction and worsen clinical outcomes. Consequently, accurate prenatal diagnosis is essential to ensure appropriate postnatal management and avoid potentially harmful treatment strategies [8].

However, the accuracy of prenatal CHD diagnosis varies considerably among examiners, and training experts in fetal echocardiography is both time-consuming and resource-intensive. To support less experienced examiners during fetal cardiac ultrasound screening, a variety of AI-based technologies have been developed, and several AI-enabled medical devices are now commercially available [35,36]. These technologies include the automated detection of standard imaging planes and identification of structural cardiac abnormalities in fetal cardiac ultrasound videos [37,38]. Despite these advances, ultrasound screening for TAPVC remains challenging because the pulmonary veins are small and are visualized only briefly during routine examinations. Several indirect sonographic findings have therefore been proposed to aid diagnosis, including the presence of a pulmonary venous confluence posterior to the left atrium, enlargement of the posterior left atrial space, and dilation of the superior vena cava or coronary sinus [39,40]. Based on these indirect findings, the PLAS index and LAPSD ratio were developed as biometric parameters for TAPVC screening.

To improve ultrasound screening for TAPVC, we developed a fully automated framework that performs the entire process, from extraction of appropriate 4CV images to calculation of the PLAS index and LAPSD ratio, in a single workflow. We first evaluated the segmentation of the heart, crux, and descending aorta using DeepLabv3+, UNet3+, and SegFormer. Among these structures, the crux proved to be the most challenging to segment accurately. The four cardiac chambers and descending aorta typically appear as hypoechoic regions, facilitating their identification and segmentation. In contrast, the crux appears as a small, hyperechoic, cross-shaped structure. Its visibility may also be reduced during diastole when the mitral and tricuspid valves are open, making precise localization difficult. To address this challenge, we expanded the segmentation region to encompass the entire cross-shaped structure and used the centroid of the segmented area for subsequent measurements. This approach represented the most practical strategy during model development and likely contributed to the lower segmentation accuracy observed for the crux compared with the other structures. Wang et al. proposed the PLAS ratio, which is calculated by drawing a line connecting the center coordinates of the epicardium (A) and descending aorta (B) on the 4CV and identifying its intersection with the epicardial border (C), thereby measuring the BC/AC ratio [41]. Although this approach does not require crux segmentation, accurate identification of the epicardial center can be challenging during routine clinical examinations. Therefore, we focused on the PLAS index and LAPSD ratio, which are more readily applicable in clinical practice. Based on the segmentation results, no substantial differences were observed between ME and AE images. Among the three segmentation models, SegFormer generally achieved the highest mDice values across case groups. However, when TAPVC screening performance was evaluated using ROC curve analysis, AE-DeepLabv3+ achieved the highest AUC values for both the PLAS index and LAPSD ratio. Overall, the AE-based methods demonstrated screening performance comparable to that of the ME-based methods, supporting the clinical feasibility of the AE approach.

Furthermore, we conducted a clinical comparison study to evaluate the performance of the AE-based methods against that of obstetricians. The AE-based methods consistently achieved higher screening accuracy than fellows and residents, with AE-SegFormer demonstrating the best overall performance among the automated approaches for both the PLAS index and LAPSD ratio. Minor differences in segmentation performance between DeepLabv3+ and SegFormer may account for the variation in the optimal model observed across different test datasets. Experts achieved exceptionally high screening performance, highlighting the substantial influence of examiner experience and expertise on TAPVC detection. For example, some forms of TAPVC are characterized by a pulmonary venous confluence that can be difficult to distinguish from the left atrium. This subtype generated the highest number of incorrect interpretations in the present clinical comparison study. Previous reports have also shown that, when the pulmonary venous confluence is located more cranially than the standard 4CV plane, LD may appear relatively narrow and the PLAS index may remain within the normal range, further complicating diagnosis [42]. In routine clinical practice, most fetal examinations are normal, and CHD is encountered relatively infrequently. TAPVC is particularly challenging because it is both rare and clinically severe, and many obstetricians may never encounter a case during their careers. Consequently, diagnosis can be difficult for examiners with limited experience or familiarity with the condition. Although further refinement and clinical validation are needed before widespread implementation, the proposed fully automated methods have the potential to support less experienced examiners, improve diagnostic consistency, and streamline the screening workflow. Ultimately, this approach may contribute to higher prenatal detection rates of TAPVC.

This study has several limitations. First, the number of TAPVC cases was limited because of the rarity of the disease. Although only normal cases were used for model training, the small number of TAPVC cases in the test dataset increases uncertainty in the evaluation of screening performance. In addition, older TAPVC cases could not be included because their ultrasound videos were acquired using earlier-generation ultrasound systems, potentially resulting in image quality that was insufficient for reliable AI analysis. Future multicenter prospective studies are therefore needed to increase the number of TAPVC cases and improve the robustness of model evaluation. Second, the sensitivity and specificity of the PLAS index and LAPSD ratio are not perfect, as reported in previous studies. Nevertheless, these indices provide an important opportunity to raise suspicion for TAPVC and should be interpreted in conjunction with other ultrasound findings rather than as standalone diagnostic tools. Third, segmentation of the crux remained challenging, and segmentation performance was lower for non-TAPVC CHD and TAPVC cases than for normal cases. Development of more advanced segmentation models capable of accounting for morphological changes in the crux during the cardiac cycle may improve performance. Since the calculation of both indices relies on the precise localization of small anatomical landmarks, further refinement and error analyses are required before clinical application. Finally, all ultrasound videos were acquired using equipment from a single vendor at a single institution. Broader clinical implementation of the proposed framework will require validation using datasets obtained from ultrasound systems manufactured by other vendors at multiple centers. This strategy may address the limitations of generalization and potential domain-shift effects.

## 5. Conclusions

We developed fully automated methods for calculating the PLAS index and LAPSD ratio for prenatal ultrasound screening of TAPVC. In the clinical comparison study, AE-DeepLabv3+, AE-UNet3+, and AE-SegFormer achieved mean AUC values of 0.903, 0.928, and 0.940, respectively, for the PLAS index, and 0.919, 0.916, and 0.940, respectively, for the LAPSD ratio. Although the screening performance of these automated methods did not match that of expert fetal echocardiographers, it consistently exceeded that of residents and fellows. As the demand for prenatal diagnosis continues to increase worldwide, technologies that support less experienced examiners may play an important role in a variety of clinical settings. For example, these methods could facilitate screening in primary and secondary care centers, enabling the identification of suspected TAPVC cases and timely referral to specialists and tertiary care institutions for further evaluation and management. Such an approach may contribute to improved clinical outcomes through earlier diagnosis and treatment planning. Our findings are still in the pilot phase owing to the abovementioned limitations. Therefore, future work will focus on improving the generalizability and clinical utility of the proposed framework. Specifically, this includes evaluating additional segmentation models, investigating workflow efficiency and inter-clinician variability, and conducting further prospective clinical validation studies with multi-center, multi-vendor, and larger TAPVC cohorts.

## Figures and Tables

**Table 1:** Segmentation performance of the three models using ME and AE 4CV images, evaluated by mDice.

		Normal Cases			Non-TAPVC CHD Cases			TAPVC Cases		
4CV Extraction	Segmentation Model	Heart	Crux	dAo	Heart	Crux	dAo	Heart	Crux	dAo
ME	DeepLabv3+	0.930	0.806	0.840	0.867	0.573	0.800	0.815	0.302	0.631
	UNet3+	0.928	0.790	0.801	0.827	0.469	0.774	0.772	0.286	0.516
	SegFormer	0.935	0.808	0.845	0.890	0.582	0.791	0.826	0.377	0.627
AE	DeepLabv3+	0.921	0.621	0.802	0.886	0.525	0.745	0.900	0.438	0.703
	UNet3+	0.920	0.590	0.751	0.853	0.470	0.700	0.855	0.398	0.700
	SegFormer	0.930	0.619	0.792	0.905	0.545	0.751	0.914	0.535	0.778

mDice, mean Dice coefficient; 4CV, four-chamber view; ME, manual extraction; AE, automated extraction; CHD, congenital heart disease; TAPVC, total anomalous pulmonary venous connection; dAo; descending aorta.

## Data Availability

The datasets generated and/or analyzed during the current study are not publicly available in order to protect participant privacy.

## Supplementary Materials

The following supporting information can be downloaded at: https://www.mdpi.com/article/10.3390/bioengineering13070822/s1. Figure S1: Schematic illustration of the programmed measurement process. Figure S2: Procedures followed by the obstetricians in the clinical comparison study. Figure S3: PR curves for each method in TAPVC screening performance. Figure S4: PR curves for obstetricians and the fully automated methods in TAPVC screening performance.

## Abbreviations

The following abbreviations are used in this manuscript:

AI	Artificial intelligence
AUC	Area under the curve
CHD	Congenital heart disease
FN	False negatives
FP	False positives
LAPSD ratio	Left-atrial posterior-space-to-diagonal ratio
mDice	Mean values of the Dice coefficient
PLAS index	Post-left atrium space index
ROC	Receiver operating characteristic
TAPVC	Total anomalous pulmonary venous connection
TP	True positives
YOLO	You only look once
4CV	Four-chamber view
