# Supplementary material for: Fully Automated Biometric Parameter Measurement in Prenatal Ultrasound Screening for Total Anomalous Pulmonary Venous Connection
Source: Bioengineering (Basel). 2026 Jul 17;13(7):822. doi: 10.3390/bioengineering13070822 (PMC13405166; doi:10.3390/bioengineering13070822)
Supplement: Supplementary file 1 [file bioengineering-13-00822-s001.zip › bioengineering-4406792-supplementary.pdf]

Supplementary information for

Fully automated biometric parameter measurement in prenatal ultrasound screening for total anomalous pulmonary venous connection

Rina Aoyama, Naoaki Harada, Masaaki Komatsu, Reina Komatsu, Katsuji Takeda, Naoki Teraya, Ken Asada, Syuzo Kaneko, Kazuki Iwamoto, Ryu Matsuoka, Akihiko Sekizawa and Ryuji Hamamoto.

The file contains
The legends of Supplementary Figures S1-4

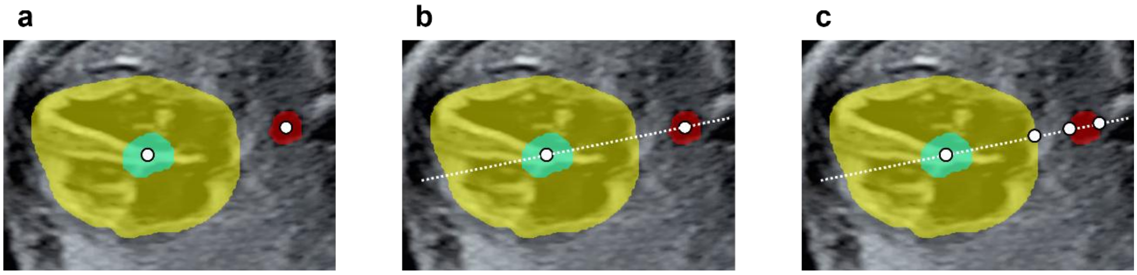

Supplementary Figure S1. Schematic illustration of the programmed measurement process. Based on the correct labels and segmentation results, the coordinates of the center of gravity of the annotated or segmented crux and descending aorta were calculated (**a**). Then, a straight line was drawn that passed through the center of gravity of the crux and descending aorta (**b**). The coordinates of where the straight line intersected with the circumference of the heart and that of the descending aorta (ventral and dorsal) were obtained. Finally, The distance was calculated from the four coordinates (**C**).

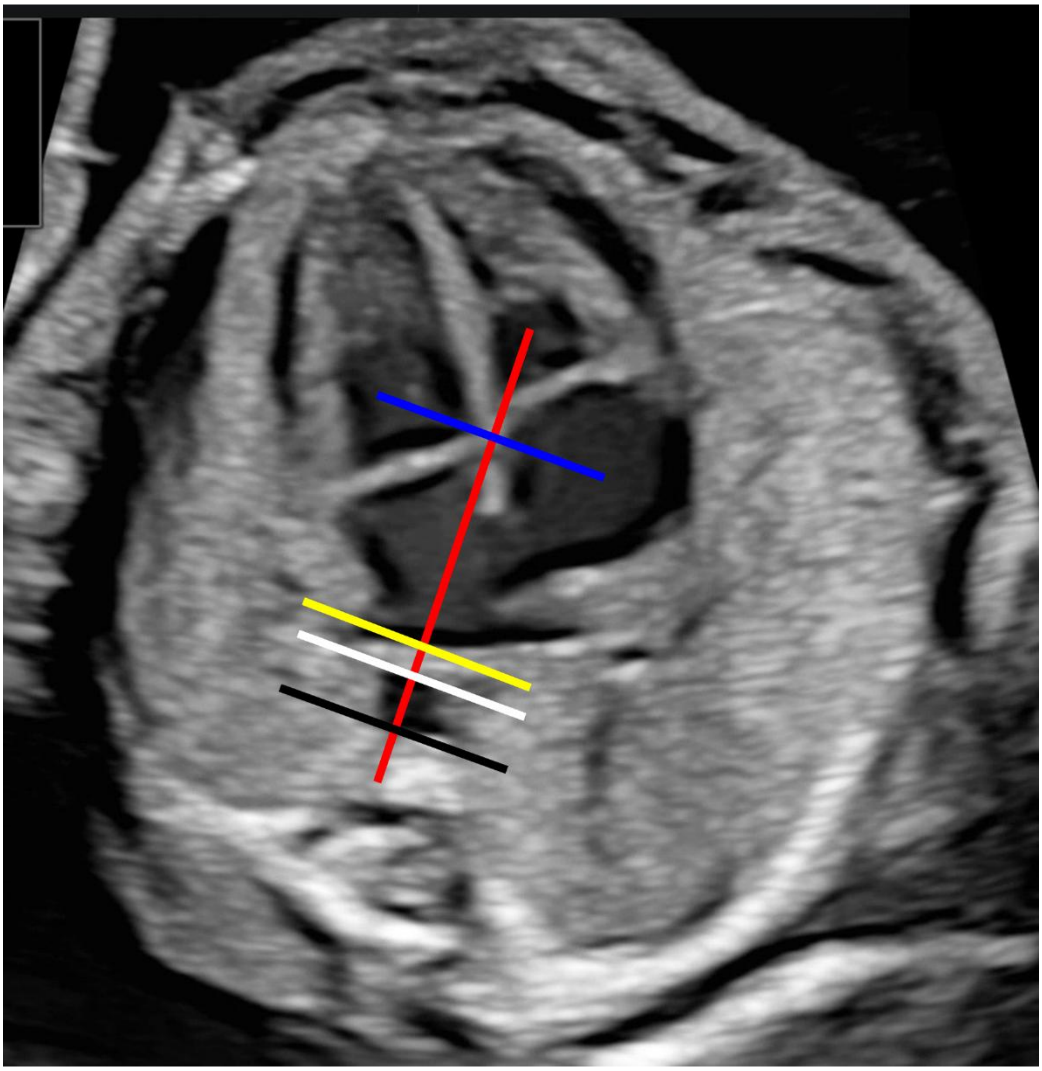

Supplementary Figure S2. Procedures followed by the obstetricians in the clinical comparison study. After manual 4CV extraction, they draw a straight red line from the crux through the descending aorta, and then draw lines perpendicular to that line at the intersection of the crux (blue), the posterior wall of the left atrium (yellow), and both ventral (white) and dorsal (black) of the descending aorta.

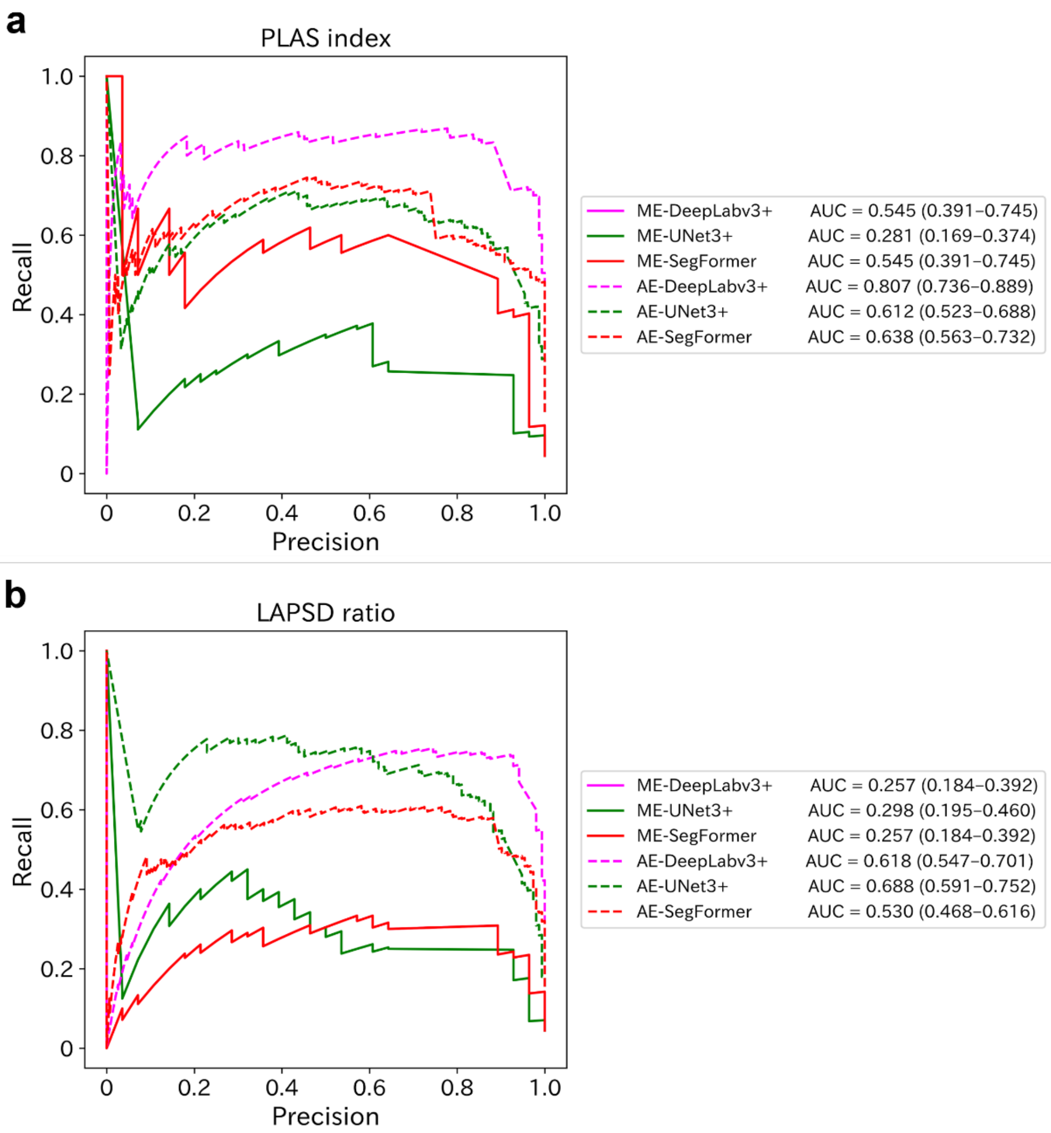

Supplementary Figure S3. PR curves for each method in TAPVC screening performance. Three segmentation models following ME and AE are shown in the PLAS index (**a**) and LAPSD ratio (**b**). Mean AUC values with 95% confidence intervals are provided in the legends. PR, precision recall; AUC, area under the precision-recall curve; AE, automated extraction; ME, manual extraction; PLAS index, post-left atrium space index; LAPSD ratio, left-atrial posterior-space-to-diagonal ratio; TAPVC, total anomalous pulmonary venous connection.

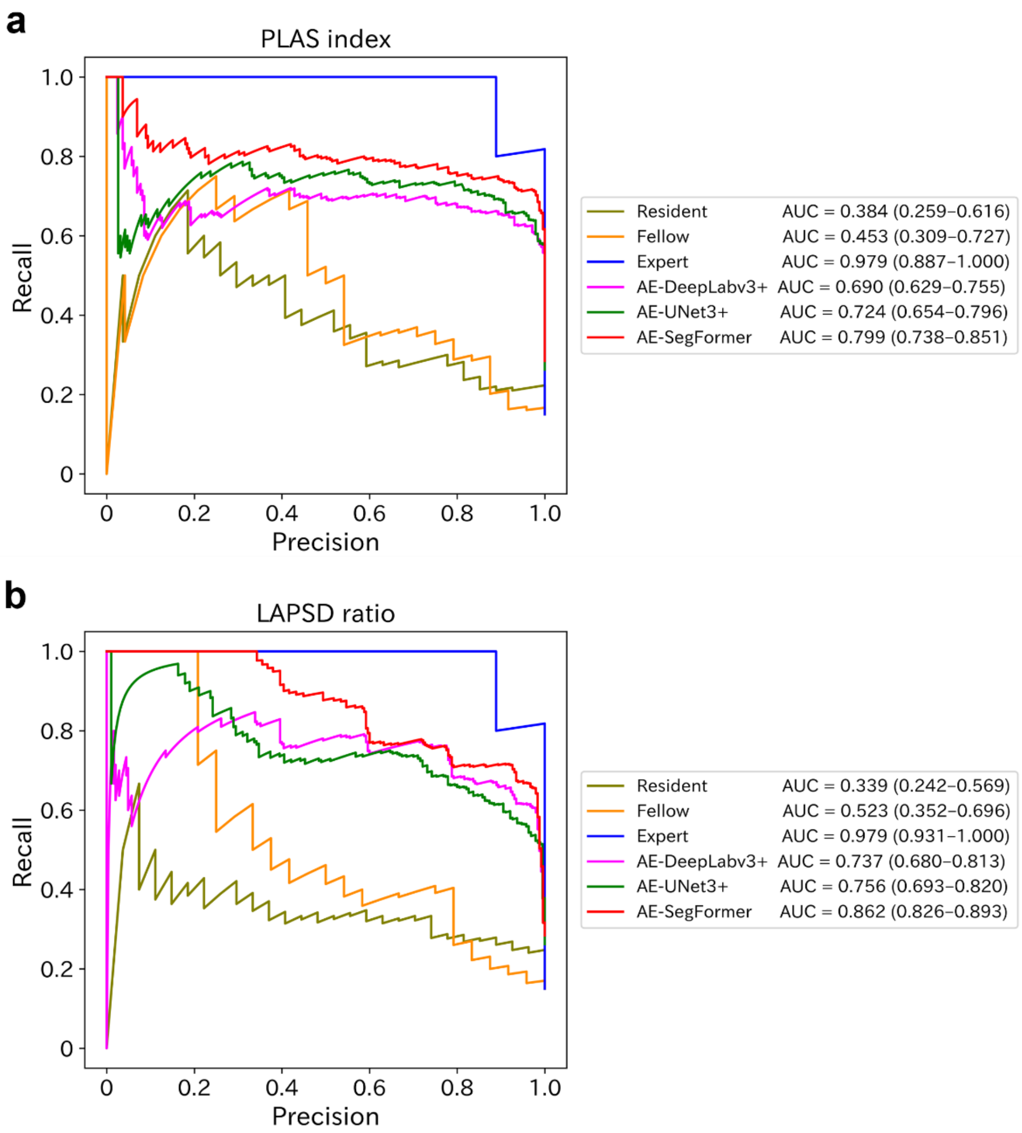

Supplementary Figure S4. PR curves for obstetricians and the fully automated methods in TAPVC screening performance. The PLAS index (**a**) and LAPSD ratio (**b**). PR curves illustrate the screening performance of experts, fellows, residents, and the fully automated methods. Mean AUC values with 95% confidence intervals are provided in the legends. PR, precision recall; AUC, area under the precision-recall curve; AE, automated extraction; PLAS index, post-left atrium space index; LAPSD ratio, left-atrial posterior-space-to-diagonal ratio; TAPVC, total anomalous pulmonary venous connection.
